# 醛类标志物的化学衍生化色谱-质谱分析方法进展

**DOI:** 10.3724/SP.J.1123.2021.02023

**Published:** 2021-08-08

**Authors:** Shuyun ZHU, Xian-En ZHAO, Huwei LIU

**Affiliations:** 1.曲阜师范大学化学与化工学院, 山东省生命有机分析重点实验室, 山东 曲阜 273165; 1. Key Laboratory of Life-Organic Analysis of Shandong Province, School of Chemistry and Chemical Engineering, Qufu Normal University, Qufu 273165, China; 2.北京大学化学与分子工程学院, 北京 100871; 2. College of Chemistry and Molecular Engineering, Peking University, Beijing 100871, China

**Keywords:** 醛, 标志物, 色谱-质谱, 衍生化, 同位素标记, 综述, aldehydes, biomarkers, chromatography-mass spectrometry, derivatization, isotope labeling, review

## Abstract

人体接触环境中的化学污染物会导致多种疾病,包括癌症、糖尿病、心血管疾病、神经退行性疾病(阿尔茨海默症、帕金森病等)等。作为一类具有高反应活性的亲电化合物,醛类(包括外源性醛类或环境污染物暴露后产生的内源性醛类)可与人体中多种重要生物分子形成共价修饰产物而产生毒害作用。暴露组研究自2005年被首次提出以来一直是一个前沿热门领域,暴露组研究可绘制生物标志物与疾病风险之间的复杂关系,因此,所有生物标志物的可测量的和特征性的变化共同构成了暴露组研究的关键基础。醛类是化学暴露组的主要成分之一。由于醛类化合物自身物理化学性质和样品大量基质干扰存在,对它们进行分析和表征特别困难。醛类化合物的分析检测方法主要有传感分析法、电化学法、荧光成像、色谱法、质谱法、色谱-质谱联用法等。基于色谱-质谱的分析技术已成为化学暴露组研究的主要方法之一。化学衍生化,特别是稳定同位素标记衍生化(亦称化学同位素标记)结合液相色谱-质谱(LC-MS)技术能够解决靶向和非靶向代谢组和暴露组分析工作中的诸多问题。化学衍生化联合色谱-质谱的分析策略是复杂体系中醛类精准分析非常重要的解决方案之一。特别是近5年,基于化学衍生化的色谱-质谱分析方法开发与应用已成为醛类分析方法中的热点和亮点。该文主要总结与评述了近5年基于化学衍生化的气相色谱-质谱(GC-MS)和LC-MS最新进展,重点关注生物基质(血液、尿液、唾液、生物组织等)中醛类暴露标志物的分析方法进展。通过探讨标记小分子醛的各种衍生试剂、定性/定量分析方法及应用价值,评述醛类暴露标志物不同分析方法的优缺点以及未来发展趋势,为暴露组学、代谢组学、脂质组学的整合发展和环境生态健康研究提供一定的帮助。为了阐明外源性和内源性醛类化合物在生理和病理事件中所起的复杂作用,需要大力改进研究醛组学(aldehydome)的分析表征技术和工具。随着更先进的质谱仪的研发和使用,以及高效色谱分离和不断进步的生物信息学手段,并同时伴随着单细胞分析、质谱成像的兴起,未来的醛类暴露组分析方法会具有更高的灵敏度、更高的分析通量,更有希望筛选鉴定未知醛类化合物并发现新的暴露组生物标志物。

## Abstract (en)

Human exposure to chemical pollutants in the environment can cause a variety of diseases, including cancer, diabetes, cardiovascular disease, and neurodegenerative diseases (Alzheimer’s disease, Parkinson’s disease, etc.). Exogenous and environmental pollutant exposure-induced endogenous aldehydes are highly reactive electrophilic compounds that can form covalently modified products with a variety of important biological molecules in the human body, thus inducing toxic effects. Exposome research has become a hotspot since it was first proposed in 2005. Exposure studies can map the complex relationships between biomarkers and disease risk. Therefore, the measurable and characteristic changes of all biomarkers together constitute a key basis for exposome research. Aldehydes are among the main components of chemical exposure. Because of the physical and chemical properties of aldehydes and the existence of multiple matrix interferences in the samples, it is particularly difficult to analyze and characterize them. The analysis and detection methods for aldehydes mainly include sensing analysis, electrochemical methods, fluorescence imaging, chromatography, mass spectrometry (MS), and chromatography-MS. Analytical techniques based on gas chromatography-MS (GC-MS) and liquid chromatography-MS (LC-MS) have emerged as the main methods for chemical exposome research. Chemical derivatization, especially stable isotope labeling derivatization (also known as chemical isotope labeling) combined with LC-MS analytical techniques, can help circumvent the problems encountered in targeted and non-targeted metabolome and exposome analysis. The combination of chemical derivatization with chromatography-MS is one of the most important solutions for the accurate analysis of aldehydes in complex samples. Over the past five years, the development and application of chromatography-MS analytical methods based on chemical derivatization have become key topics in aldehyde analysis. This paper summarizes and reviews the latest progress in GC-MS and LC-MS methods based on chemical derivatization (2015-2020). The review focuses on analytical method development for aldehyde exposure biomarkers in bio-matrices (blood, urine, saliva, biological tissue, etc.). Various derivatization reagents for labeling small-molecule aldehydes, qualitative/quantitative analytical methods and their application value, advantages/disadvantages of different analytical methods for aldehyde exposure biomarkers, and future development trends are also included. The manuscript contents may aid the integrated development of exposome, metabolomics, and lipidomics, as well as research on the environment, ecology, and health. To clarify the complex actions of exogenous and endogenous aldehydes in physiological and pathological events, it is necessary to improve the analysis and characterization techniques and tools for studying the “aldehydome.” With the development and application of sophisticated mass spectrometers, advances in high-performance chromatographic separation and bioinformatics, and advent of single-cell analysis and MS imaging, future aldehyde exposome analytical methods will have higher sensitivity and throughput. This in turn would be more useful for screening and identifying unknown aldehyde compounds and discovering new exposome biomarkers.

人类的许多疾病与暴露于环境中的大量化学物质有关。人体接触环境中的化学污染物会导致多种疾病,包括癌症、糖尿病、心血管疾病、神经退行性疾病(阿尔茨海默症、帕金森病等)等。醛类是高反应活性的亲电化合物,可与人体多种生物分子(如磷脂、肽、调节蛋白、酶和DNA)形成共价修饰产物而产生毒害作用。2005年,Christopher Wild^[1]^首次提出“暴露组”概念,以解释环境暴露在人类疾病发病机制中的作用。自诞生以来,暴露组研究一直是一个前沿热门领域。化学暴露组(chemical exposome)是指人们从胚胎开始就接触到的化学污染物的总合。研究表明,暴露组研究可绘制生物标志物与疾病风险之间的复杂关系,因此,所有生物标志物的可测量的和特征性的变化共同构成了暴露组研究的关键基础^[2,3,4,5,6]^。

醛类是化学暴露组的主要成分之一,在多种疾病的发病机理中属于重要因素之一。醛暴露组(aldehydome)包括所有内源性和外源性醛的总和,一方面是生命体在生理、病理、环境污染物急性损伤等状态下产生的内源性醛类,另一方面来源于外源性醛类污染物^[7,8]^。甲醛、乙醛、丙二醛(MDA)等就是人体生理或病理状态下的重要醛类化合物。近期,Semerad等^[9]^监测到纳米零价铁暴露的微生物活体体系能够产生6种醛类损伤标志物:甲醛、丙烯醛、甲硫基丙醛、苯甲醛、乙二醛和甲基乙二醛,并且6种醛含量均显示出与纳米污染物剂量的相关性。环境污染物双酚A(BPA)暴露会诱发人体氧化应激,显著升高人体尿液中的MDA含量^[10]^,是导致神经系统或生殖发育系统等疾病的重要诱因^[11,12]^。甲醛是中国乃至全世界典型的外源性醛类污染物,能够导致神经毒性、遗传毒性、癌症等^[13]^。

由于醛类化合物自身物理化学性质和样品大量基质干扰存在,对它们进行分析和表征特别困难。醛类化合物的分析检测方法主要有传感分析法、电化学法、荧光成像、色谱法、质谱法、色谱-质谱联用法等。基于质谱的分析技术已广泛用于生物分析和环境监测研究中,并已成为化学暴露组研究的主要方法之一^[14,15]^。超高效液相色谱-串联质谱(UHPLC-MS/MS)或气相色谱-串联质谱(GC-MS/MS)技术,由于其灵敏度高、特异性强、动态范围宽的优势,已成为直接测量生物或环境样品中化学物质(包括外源性物质、其代谢产物和生物标志物)最常用的方法。暴露组质谱分析方法主要包括靶向分析法(targeted analysis)和非靶向发现法(non-targeted profiling)。对于靶向方法,通过复杂的样品前处理净化步骤和使用昂贵的同位素内标,往往能精准测量一类或几类已知化学物质。非靶向方法通过牺牲灵敏度来保留广泛的分析物覆盖范围,使该方法在测量低浓度化学物质方面效率降低^[5]^。化学衍生化(chemical derivatization),特别是稳定同位素标记衍生化/化学同位素标记(stable isotope labeling derivatization or chemical isotope labeling)能够解决代谢组和暴露组LC-MS靶向和非靶向分析工作中的诸多问题^[2,16-19]^,不但能够使质谱检测灵敏度提高10~1000倍之多,而且在提高样品前处理效果、改善色谱分离、增强MS离子化、拓宽动态范围、提高MS/MS碎裂特异性、发现新代谢物和标志物等方面具有显著优势。

关于醛的分析检测进展综述,Berdyshev^[20]^在2011年评述了脂肪醛的GC-MS和LC-MS分析方法进展,Jha等^[21]^在2017年、Laghrib等^[22]^在2019年分别评述了醛类传感分析相关进展,2019年Kishikawa等^[23]^综述了生物/食品/环境样品中醛的样品前处理技术和基于色谱的分析方法进展,Dator等^[24]^综述了生物样本中醛类的生物分析和质谱分析方法。化学衍生化联合色谱-质谱的分析策略是复杂体系中醛类精准分析非常重要的解决方案之一,特别是近5年,基于化学衍生化的色谱-质谱分析方法开发与应用已成为醛类分析方法中的热点和亮点。本文主要总结与评述了近5年来基于化学衍生化的GC-MS和LC-MS用于生物基质(重点关注血液、尿液、唾液、脑脊液、生物组织等)中醛类标志物的最新分析方法进展。未见其他色谱-质谱联用技术(包括毛细管电泳、超临界流体色谱、微流控)用于醛类分析的工作,对于食品、水土气等样本以及醛类的传感分析法、电化学法、荧光成像、常规色谱法(主要包括基于衍生化的HPLC-UV、HPLC-FLD、GC-FID 3类方法)也不再进行评论。本文通过探讨标记小分子醛的各种衍生试剂、定性/定量分析方法及应用价值,评述醛类暴露标志物不同分析方法的优缺点以及未来发展趋势,为暴露组学、代谢组学、脂质组学的整合发展和环境生态健康研究提供一定的帮助。

## 1 气相色谱-质谱法

GC-MS已广泛用于醛类分析。对于挥发性醛,可以结合顶空(HS)萃取直接进行GC分析,无需衍生化。然而,大量报道的方法是利用衍生化来改善醛的挥发性、稳定性和电离效率^[23]^。与LC或LC-MS广泛使用的2,4-二硝基苯肼(DNPH)衍生试剂不同,在GC或GC-MS醛类分析中,苯环上含有氯、氟的肼/胺类衍生试剂具有显著优势。例如常用2,4,6-三氯苯肼(TCPH)、五氟苯肼(PFPH)、2,3,4,5,6-五氟苯羟胺(PFBHA)等衍生试剂,它们能够更好地提高衍生物的挥发性和热稳定性,同时能够生产更稳定和特异的检测离子。2016年,Serrano等^[25]^报道了一种基于PFBHA衍生化的HS-GC-MS分析方法,实现了人尿液中12种内源性醛类的定性和定量测定,可用于吸烟人群或糖尿病患者醛类标志物监测。2019年,Wei等^[26]^采用PFBHA衍生化法建立了人类血液中7种小分子醛类代谢物(C_1_~C_7_)的HS-GC-MS分析方法。对膀胱癌患者(*n*=15)和正常人群组(*n*=15)的测定和统计分析(T检验)结果表明,7种脂肪醛含量在两组之间均具有显著差异,而且其中6种脂肪醛在膀胱癌患者血液中的含量极显著性升高(*P*<0.001,丁醛除外)。小分子醛类代谢物具有生物标志物的巨大潜力,衍生化技术能够显著改善GC-MS检测的灵敏度、选择性、准确度等,在标志物筛选、疾病筛查以及临床研究等方面具有良好的科学价值。

近期,Semerad等^[9]^采用PFBHA衍生化HS-SPME-GC-MS分析方法,监测细菌、真菌和藻类培养基受到纳米零价铁暴露损伤产生的醛类标志物,结果表明能够检测到微生物活体体系产生的6种醛类化合物(甲醛、丙烯醛、甲硫基丙醛、苯甲醛、乙二醛和甲基乙二醛),并且6种醛含量均显示出与纳米污染物剂量的相关性。该文作者认为这可能是第一篇研究微生物暴露于铁纳米颗粒后的氧化应激状态产生醛类标志物的文献^[9]^。

## 2 液相色谱-质谱法

### 2.1 常规衍生化LC-MS/MS分析

具有串联质谱功能的LC-MS/MS已成为LC-MS分析的主流,这是因为MS/MS中多反应监测(MRM)模式的检测特异性得到了显著提高,可减少干扰并提供快速、多通道分析。由于分析物的电离效率极大地影响了MS仪器灵敏度,因此在LC-MS/MS中,衍生化技术经常用于提高醛的低电离效率。LC-MS的衍生化试剂通常具有较大的电子亲和势或者带有分子内正电荷。已报道的HPLC-UV和-FLD方法的衍生化试剂,由于其分子结构具有易于电离的部分,大多可以用于LC-MS。DNPH是一种具有代表性的基于肼反应基团的衍生试剂,已广泛应用于多种醛的衍生化LC-MS分析。另外常常使用的衍生化试剂还包括:丹磺酰肼(DnsHz)、邻苯二胺、D-半胱氨酸、9,10-菲醌(PQ)、3-硝基苯肼和3,4-二氨基二苯甲酮^[23,24,27]^,限于本综述在前言部分所述的综述范围,此处不再赘述。下面讨论近5年具有一定代表性的工作。2015年,Fernandez-Molina等^[28]^采用DNPH作为衍生试剂,开发了一种将衍生化、固相微萃取(μ-SPE)与LC-MS联合使用的快速、灵敏的分析策略,通过测定暴露人员尿液中脂肪族和芳香族低分子量醛类化合物含量(ng/L水平),可以获得这些醛类化合物在暴露人员体内的半衰期信息。冯钰锜课题组^[29]^开发了一种基于磁性固相萃取结合DNPH原位衍生化的样品前处理方法,用于测定肺癌患者尿液中的己醛和庚醛。2016年,Sobsey等^[30]^采用3-硝基苯肼为衍生试剂,开发了一种人类血浆中标志物丙二醛的LC-MS/MS检测方法,引入同位素标记的^13^C_6_-3-硝基苯肼-丙二醛衍生物作为内标,显著提高了定量的灵敏度、准确度、特异性,而且分析速度快(每样2.5 min)。2021年,Yang等^[31]^采用3-三氟甲基嘧啶肼为衍生化试剂,开发了一种大鼠脑组织中一种毒性中间体代谢物3,4-二羟基苯乙醛的LC-MS/MS分析方法,通过控制生物样品pH值和衍生化反应,生成了稳定的衍生产物,该分析方法可用于不同状态下活性醛的测定,对帕金森病等疾病相关研究具有重要意义。

郭寅龙课题组^[32]^巧妙地设计衍生化反应,采用商品化的吡啶和亚硫酰氯对脂肪醛进行季铵化衍生反应,如图1所示。衍生产物具有很强的质谱响应和特异的子离子碎片,过量的吡啶对衍生产物的干扰相对较小,过量的亚硫酰氯通过加入去离子水即可除去。通过该检测方法,在人类甲状腺组织中检测到了13种游离脂肪醛(C_6_~C_18_),其中长链脂肪醛(C_10_~C_18_)在甲状腺癌组织中的浓度显著高于癌旁组织中的浓度。更进一步地,该课题组^[33]^将该衍生反应的优势进行拓展,实现了甲状腺癌组织中脂肪醛、脂肪醇、甾醇的同时衍生和快速的离子淌度质谱测定,具有快速简便、灵敏度和准确度高等优势。

**图1:**
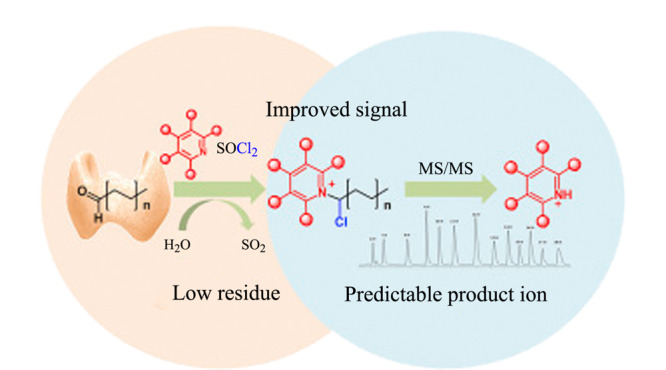
人甲状腺组织中脂肪醛的季铵化增敏衍生与MS/MS特异性检测^[32]^

醛类标志物的柱后衍生化也是一种可行的解决方案^[34]^。近期,冯钰锜课题组^[35]^采用亚硫酸铵作为柱后衍生试剂实现了醛类化合物的在线衍生和LC-MS/MS分析,具有快速简便的显著优势(见图2)。衍生产物采用负离子模式检测,在较低能量下的碰撞诱导解离即可产生特异的亚硫酸氢根产物离子,己醛和庚醛的检出限分别为3 nmol/L和2 nmol/L,而且能够实现人类尿液或中草药提取物中醛类的MRM定量测定和母离子扫描模式下的未知醛类筛选。

**图2:**
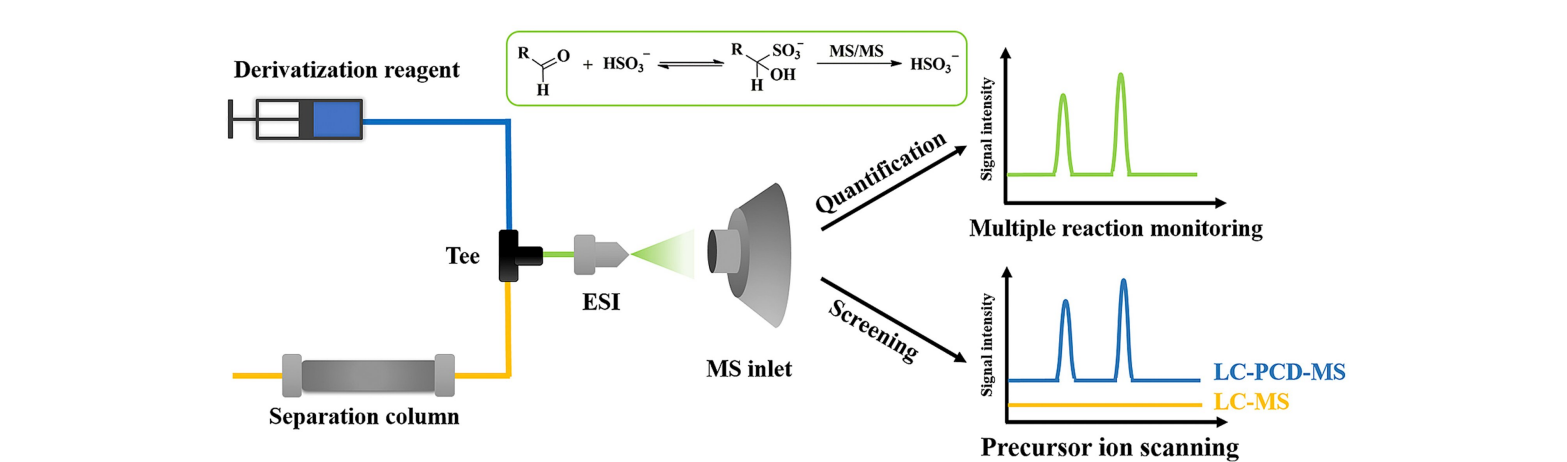
醛的柱后衍生与MRM定量分析和母离子扫描非靶向筛选^[35]^

### 2.2 化学同位素标记LC-MS/MS靶向分析

外标定量法经常受到质谱仪信号波动的影响,往往导致定量不准。加入内标进行辅助定量可以起到良好的校正效果。但内标的选择往往是个难题,几乎难以找到符合色谱分离、质谱响应定性/定量要求的内标化合物。同位素内标能够解决上述问题,但是,常规实验室难以合成符合要求的同位素内标物;而可以购买的同位素内标价格昂贵,并且很多分析物包括未知的代谢中间体、微量代谢产物等没有商品化的同位素内标。通过合成高纯的同位素标记衍生试剂,对标准品或代谢组学混合样品进行衍生制取同位素内标物,可用于LC-MS的靶向定量测定、代谢差异分析、非靶向扫描等,是目前组学分析领域的一项前沿和关键技术^[18,36-39]^。

2016年,Tie等^[40]^采用轻/重同位素标记衍生化技术,建立了内源性脂肪醛代谢物的LC-MS/MS靶向分析和扫描方法。采用H/D同位素取代的2,4-二(二乙氨基)-6-肼-1,3,5-三嗪为衍生试剂,大鼠血浆和脑组织中脂肪醛的衍生反应在37 ℃下15 min内完成,MRM定量检出限在0.1~1 pg/mL;该策略应用于颈侧动脉痴呆大鼠模型与正常组大鼠的对比研究,发现两组大鼠血浆和脑组织中分别有43种和19种脂肪醛含量发生显著变化,这表明内源性脂肪醛代谢物在活体动物生理病理研究中具有重要作用。

2018年,Kuroda研究组^[41]^在9,10-菲醌作衍生试剂的基础上,通过^14^N/^15^N-乙酸铵引入同位素N原子,实现了醛类化合物的轻/重同位素标记衍生化,从而建立了4-羟基-2-壬烯醛(HNE)和4-羟基-2-己烯醛(HHE)两种醛类标志物的LC-MS/MS靶向分析方法,该方法对人类血浆中的HNE和HHE的定量限分别达到了0.2 nmol/L和0.05 nmol/L。该方法通过血浆标志物HNE和HHE检测能够区分正常人与糖尿病、风湿病和心脏病患者。

2020年,我们研究组^[42]^设计、合成了一系列基于左氧氟沙星酰肼的质量标签(LHMTs)试剂:LHMT359/360/361/362/363/364/365/366/373/375/376/378/379/381(见图3),用于肺癌标志物己醛和庚醛的高通量LC-MS/MS靶向分析。为了增加LHMTs试剂的通量,首先设计、合成氘代的D_3_-左氧氟沙星,然后对H_3_/D_3_-左氧氟沙星进行简单的同位素形态的烷基化、酰肼化简单、高效的合成和纯化,即可得到14种LHMTs衍生试剂。如图4所示,LHMT359用于标记己醛和庚醛混标,所得衍生产物作为LC-MS/MS MRM定量分析的内标;LHMT373-庚醛衍生物用作虚拟模板,设计、制备了一种磁性分子印迹聚合物,可用于12重(12-plex)实际血清样品标记溶液与LHMT359内标溶液的混合液的磁性分散固相萃取,实现混合液中所有LHMTs-己醛/庚醛衍生物的同时、高选择性、高通量富集与净化。这种以质量差异标签为虚拟模板设计、制备的虚拟模板分子印迹聚合物既保留了分子印迹材料的独特选择性,又避免了长期以来困扰分子印迹材料样品前处理中模板泄露会导致定量不准的技术难题。一次UHPLC-MS/MS进样分析(2 min)即可实现12个血清样品的同时定量检测,己醛和庚醛的检出限为0.5 pmol/L。对肺癌患者、吸烟者和正常对照组血清检测结果表明,肺癌患者血清中己醛与庚醛的浓度显著高于正常人和吸烟者含量,因此,己醛和庚醛具有成为肺癌疾病标志物的巨大潜力。多标签化学同位素标记(MTCIL)技术是LC-MS/MS分析领域兴起的一种兼具高通量、高灵敏度、高准确度、高选择性的新策略,具有巨大优势和发展前景。在轻/重同位素标记LC-MS/MS定量分析的基础上,该技术显著提高了分析通量,更大程度发挥了MRM多通道的独特优势,将来在高通量定量和相对定量方面必将显现出更大优势。

**图3:**
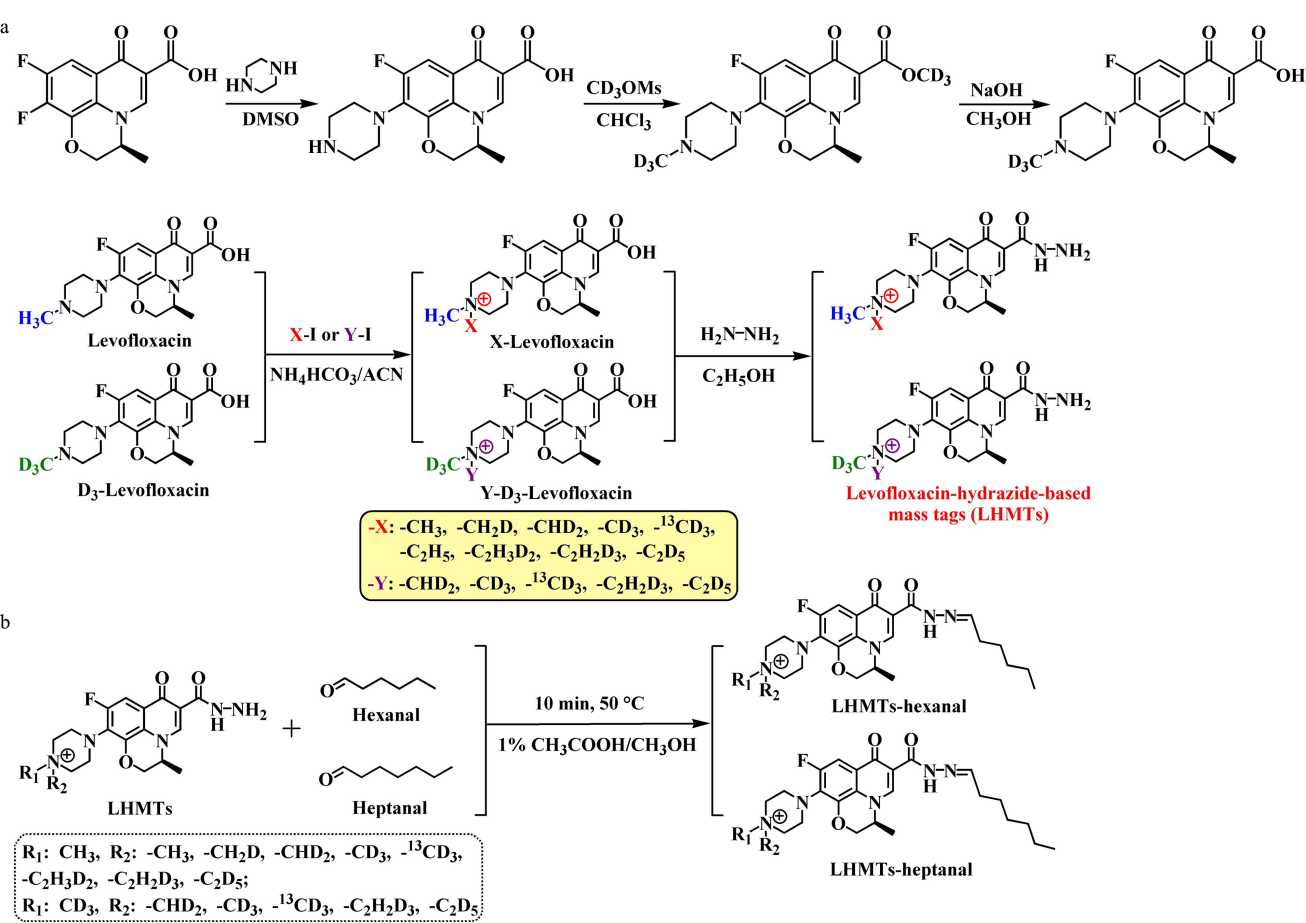
(a)14重LHMTs试剂的设计、合成以及(b)MTCIL的衍生化条件^[42]^

**图4:**
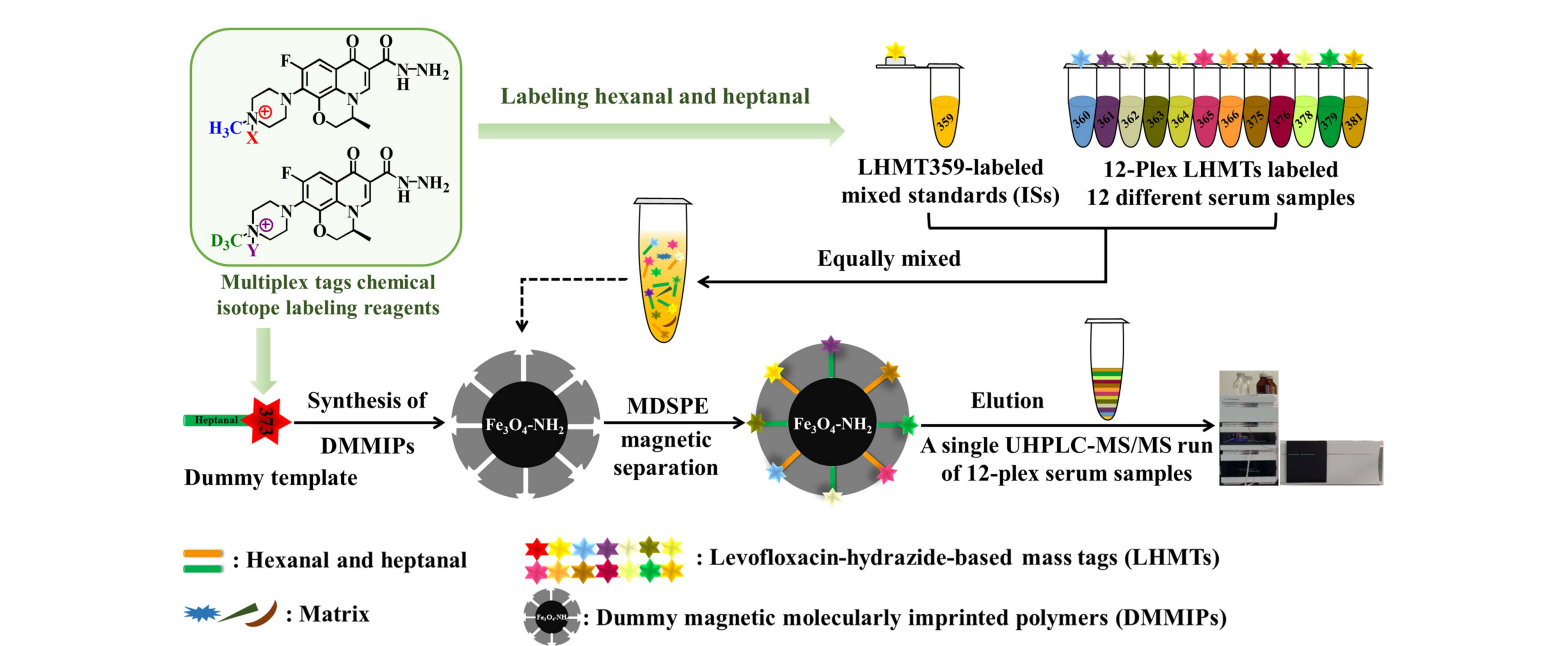
多标签化学同位素标记分析策略流程及其14种LHMTs质量标签作用^[42]^

### 2.3 化学同位素标记LC-MS非靶向扫描与相对定量

常规衍生化或化学同位素标记技术为LC-MS/MS定量分析提供了出色的灵敏度和良好的特异性,但缺乏筛选未知醛的能力。组学分析面临从大量生物样本中进行非靶向扫描、筛选生物标志物的巨大需求。化学同位素标记技术联合LC-MS的多种扫描模式(MRM、中性丢失扫描、母离子扫描、子离子扫描、全扫描等),能够将海量的样品分析物分成羰基、羧基、羟基、氨基等亚组,显著提高非靶向扫描的灵敏度、覆盖度、准确度等,是组学分析中的前沿热点^[37]^。

冯钰锜课题组^[43]^开发了一种基于轻/重同位素标记联合液相色谱-双中性丢失质谱扫描的非靶向醛类化合物扫描方法,设计、合成了一对轻/重稳定同位素标记试剂:D_0_/D_4_-4-(2-(三甲铵基)乙氧基)-苯胺溴鎓(4-APC-d_0_/d_4_),分别用于标记实际样品和合并的对照样品;在质谱碰撞诱导解离作用下,4-APC-d_0_标记和4-APC-d_4_标记的羰基化合物分别产生质量数为87 Da和91 Da的中性丢失碎片,从而通过双中性丢失扫描得到人尿液和白葡萄酒中16种和19种醛类化合物;进一步通过与合成标准品进行比较,确定了人尿中的5种和白葡萄酒中的4种醛类化合物。另外,他们^[44]^还开发了一种基于轻/重同位素标记和液相色谱-双母离子扫描质谱分析(IL-LC-DPIS-MS)策略,用于人血清中的羰基化合物鉴定和相对定量;合成了含有碳酰肼的4种不同衍生试剂,并优化选择了一对轻/重同位素标记试剂(2-(2-肼-2-羰乙基)异喹啉溴鎓(d_0_/d_7_-HIQB,见图5),分别用于标记实际血清样品和合并对照血清样品。基于d_0_/d_7_-HIQB羰基衍生物在母离子扫描模式下分别产生特征产物离子*m/z* 130.1/137.1,建立了IL-LC-DPIS-MS策略,并用于扫描鉴定人血清中潜在羰基代谢物和已知羰基代谢物的相对定量分析(见图6)。该研究总共在人血清中检测到156种候选羰基化合物(重要醛类包括:丁烯醛、异戊醛、庚醛、辛醛、壬醛、癸醛),其中12种通过合成标准品得到了进一步鉴定。与健康对照组相比,骨髓性白血病患者中有44个羰基代谢物存在统计学差异。

**图5:**
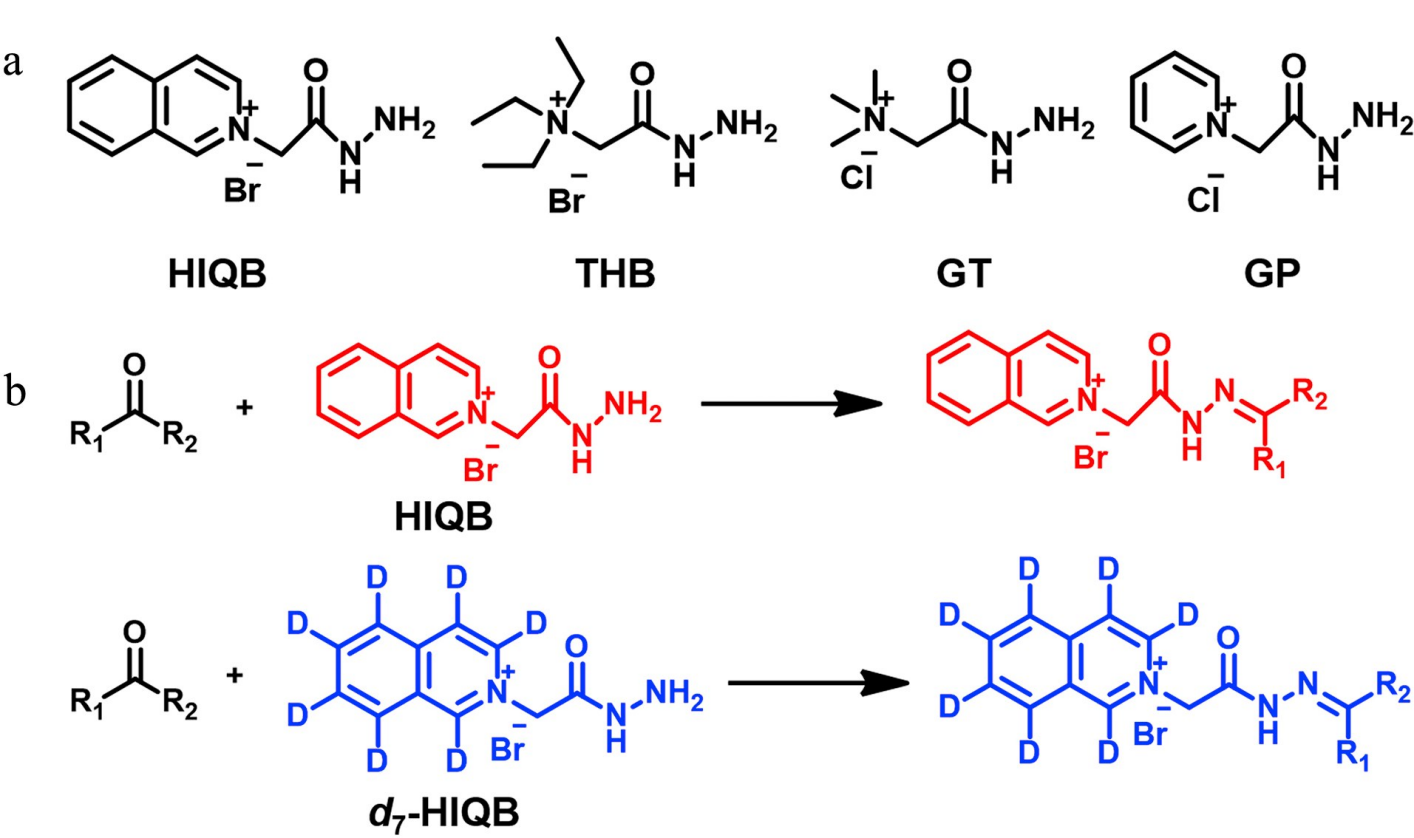
(a)4种标记试剂的化学结构和(b)d_0_/d_7_-HIQB对 羰基化合物的标记^[44]^

**图6:**
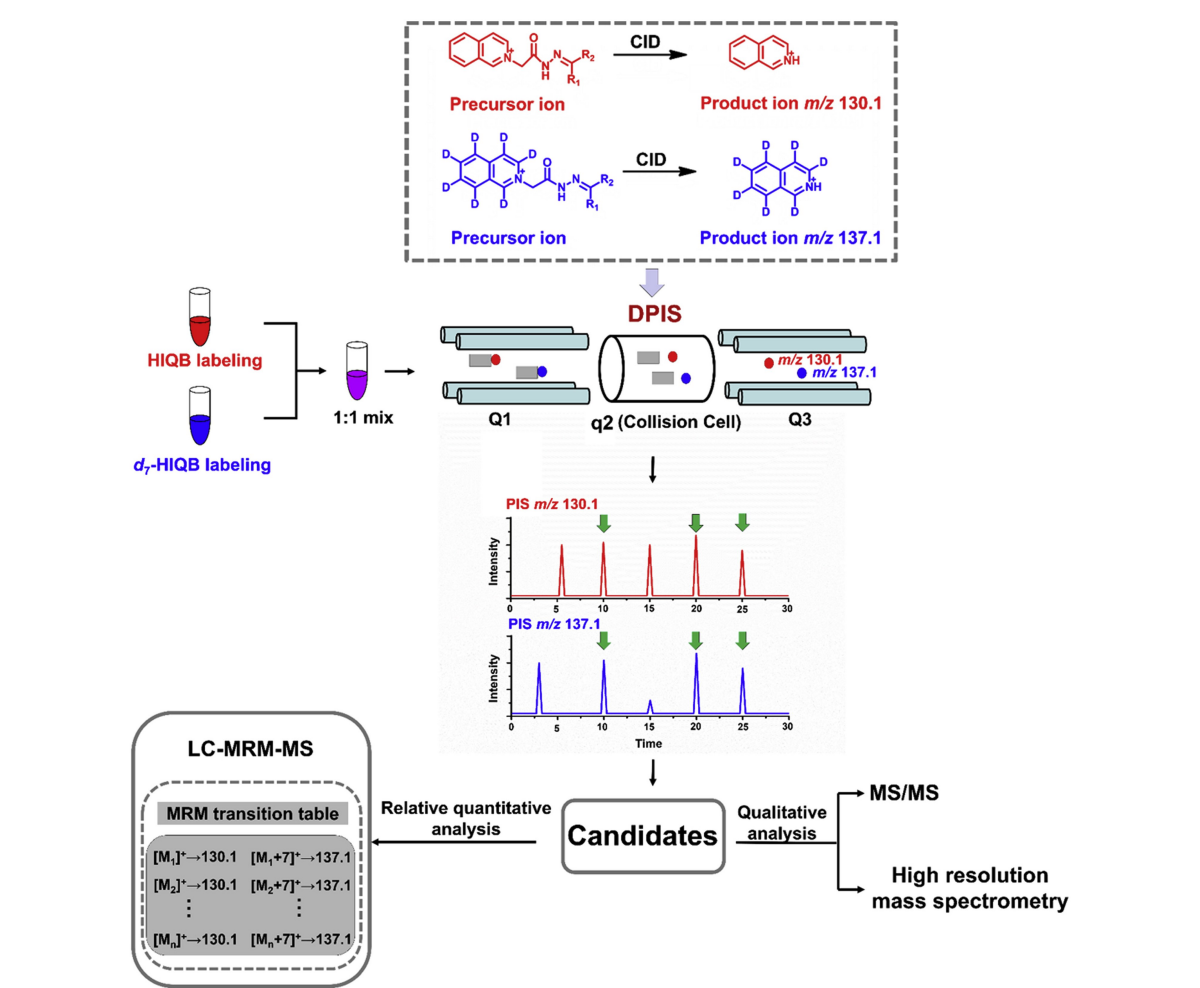
LC-DPIS-MS策略用于羰基化合物轮廓分析^[44]^

加拿大Li教授课题组^[45]^开展了一系列创新性的基于化学同位素标记的代谢组学研究工作,在羰基化合物代谢亚组的分析表征方面开发了一种高效的基于化学同位素标记的LC-MS代谢组学解决方案(CIL-LC-MS)。该解决方案以其合成的轻/重同位素标记试剂^12^C/^13^C-丹磺酰肼(^12^C/^13^C-DnsHz)为标记试剂,分别用于标记单个尿样和合并对照尿样,再采用液相色谱-四极杆飞行时间质谱(LC-QTOF-MS)羰基化合物鉴定和相对定量(见图7);开发了用于处理CIL-LC-MS质谱数据的内部软件程序,并构建了基于DnsHz标记物的数据库(www.mycompoundid.org)从而更方便地鉴定羰基代谢物。该研究总共在人类尿液中检测到1737个峰对,其中33个得到了确认。这些结果表明,^12^C/^13^C-DnsHz标记CIL-LC-MS方案是一种能够高覆盖率分析复杂样品羰基亚代谢组的有效解决手段。该课题组^[46]^进一步提高了代谢组覆盖率,通过对4个亚组的代谢物羟基、胺和酚、羧基、羰基分别进行同位素标记和LC-MS分析,在人类血浆和酵母菌中都发现了高覆盖度的峰对,这对代谢组的分析方法发展和应用提供了良好的解决方案。

**图7:**
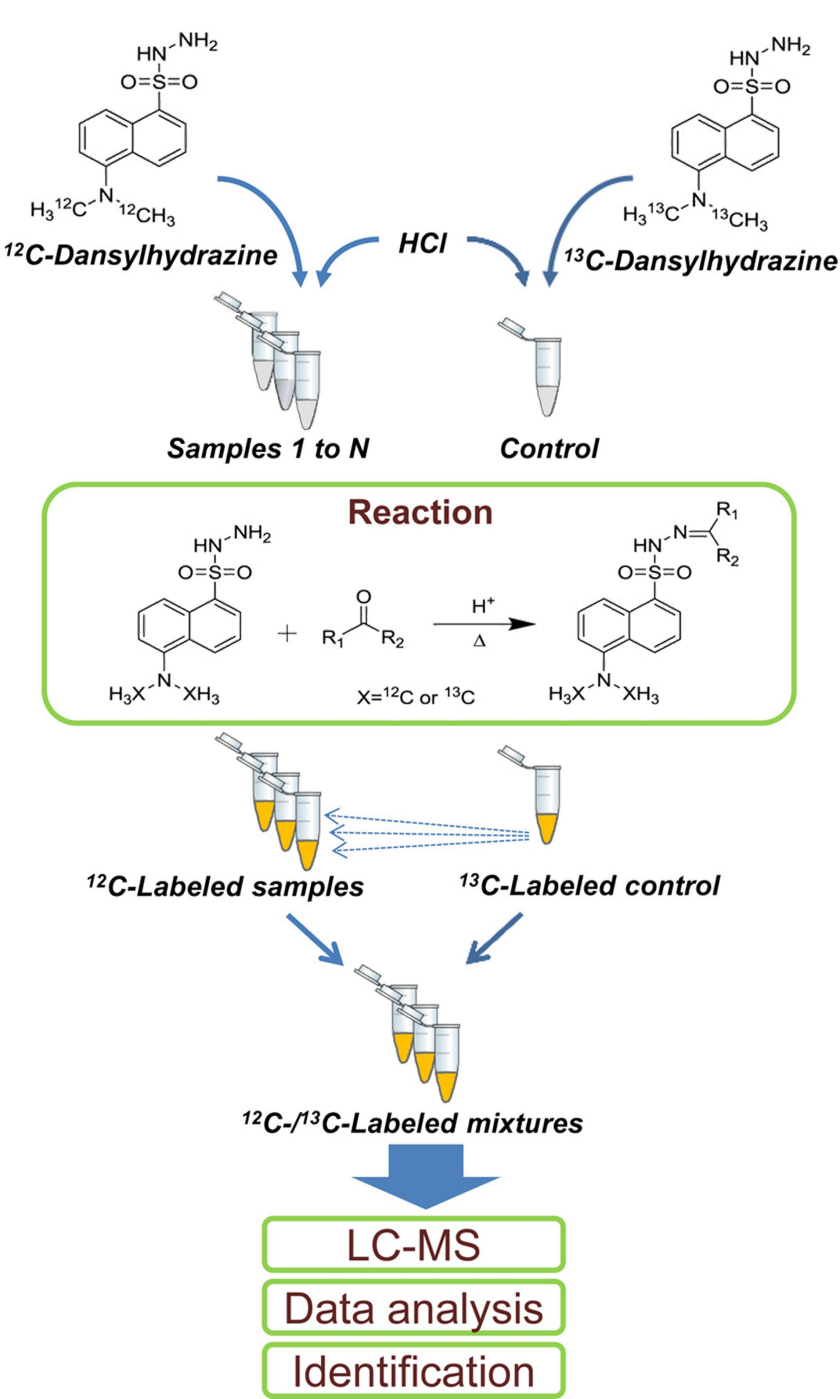
^12^C/^13^C-DnsHz标记CIL-LC-MS羰基代谢亚组相对定量分析流程^[45]^

### 2.4 衍生化液相色谱-高分辨质谱分析

液相色谱-高分辨质谱(LC-HRMS)已成为LC-MS分析领域未来重要发展方向。基于高分辨率质谱的代谢组学分析方法可提供母离子和MS/MS碎片离子的准确质量,因此可以可靠地鉴定复杂生物基质中检测到的代谢物。最近,Balbo课题组^[47]^开发了一种基于高分辨质谱的精确质量数MS^3^中性丢失扫描策略,以表征生物样本中DNPH衍生的羰基化合物,从而实现了同时检测和定量可疑和未知的羰基化合物。以前对DNPH羰基化合物衍生物的分析大多以负离子模式和相对较高的流速下进行,这限制了痕量分析物检测和定量的灵敏度。在正离子模式下,这些化合物在碰撞诱导解离后显示出特征性的羟基自由基(·OH)中性丢失。在负离子模式下未观察到这种中性丢失现象。因此,DNPH羰基化合物衍生物的特征性·OH中性丢失被用作MS数据采集扫描方法,从而可以更准确鉴定羰基化合物(见图8)。此外,通过使用D_0_/D_3_-DNPH轻/重同位素标记的相对定量策略,测定乙醇暴露后羰基化合物的相对含量。将暴露前的样品用D_0_-DNPH标记,而暴露后的样品用D_3_-DNPH标记,两种标记样品以1∶1(v/v)的比例混合,采用上述建立的方法进行分析。该分析流程提供了一种更加准确、快速、稳健的方法,可以鉴定和定量各种生物基质中的毒性羰基化合物,以进行暴露风险评估。这与以前报道的工作形成鲜明对比,之前的工作使用了相对较高的流速(0.2~1.5 mL/min)和低分辨率MS分析,从而限制了它们在痕量分析物水平上的灵敏度和鉴定可信度。Balbo课题组^[47]^应用新开发的方法来表征人类饮酒后唾液中羰基化合物的变化水平,结果表明乙醛水平在暴露后会增加。该策略亦可用于表征电子烟使用以及吸烟相关的羰基化合物暴露评估。

**图8:**
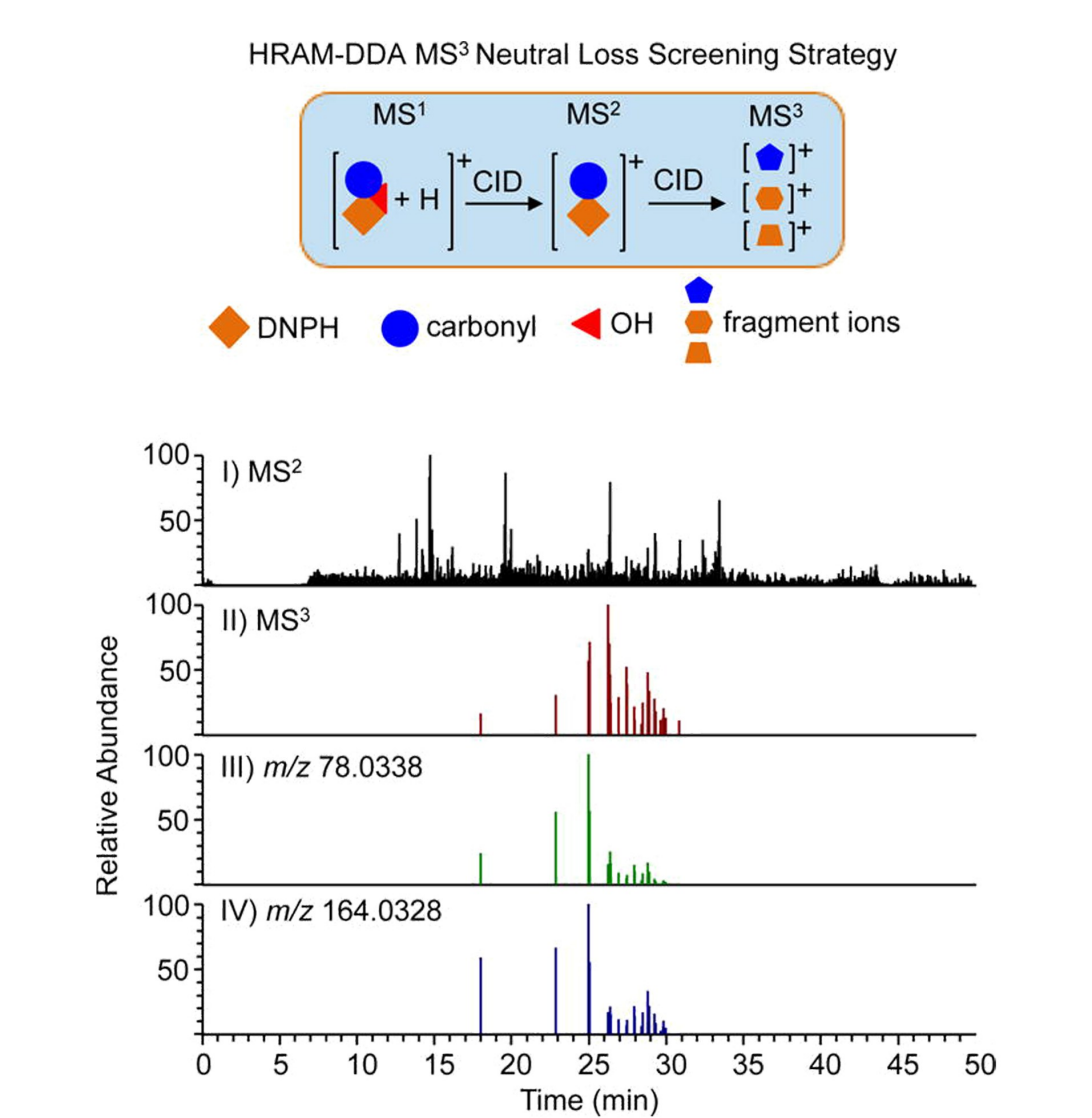
高分辨精确质量数MS^3^中性丢失扫描策略^[47]^

## 3 结论与展望

总体而言,色谱-质谱技术相结合是强大的分离分析工具,可用于筛查鉴定和定量测定各种生物基质中的醛类化合物。这些分析方法的高选择性和特异性联合从MS和MS*^n^*质谱数据获得的结构信息是鉴定和定量已知和未知化合物的理想手段。本文介绍的基于色谱-质谱联用的醛类分析方法为复杂生物基质中醛类的检测提供了更高的灵敏度、选择性和特异性。尽管这些技术非常灵敏,但它们也容易受到基质干扰的影响,需要严格的样品净化处理。另外,这些技术需要昂贵的仪器和训练有素的操作人员,并且便携性较差。基于质谱的新技术的开发正在不断向新的应用发展,特别是适合人体暴露评估的痕量分析,以此阐明它们对人类健康的贡献和影响。

醛是暴露组的主要成分,往往是在多种醛类痕量水平上,并且在某些情况下是由于内源性和外源性联合产生的。醛在包括癌症在内的多种疾病的发病机理中占据重要位置。由于它们的反应性以及多种化合物的共存性,对醛类化合物进行分析和表征特别困难。为了阐明这些外源性和内源性醛类化合物在生理和病理事件中所起的复杂作用,需要大力改进研究醛组学的分析表征技术和工具。随着更先进的质谱仪的研发和使用,以及高效色谱分离和不断进步的生物信息学手段,并同时伴随着单细胞分析、质谱成像的兴起,未来的醛类暴露组分析方法会具有更高的灵敏度、更高的分析通量,更有希望筛选鉴定未知醛类化合物并发现新的暴露组生物标志物。
